# Hepatic resection using intraoperative ultrasound and near-infrared imaging with indocyanine green fluorescence detects hepatic metastases from gastric cancer: A case report

**DOI:** 10.1016/j.ijscr.2022.106791

**Published:** 2022-01-24

**Authors:** Takeshi Tadokoro, Hiroyuki Tahara, Shintaro Kuroda, Tsuyoshi Kobayashi, Kazuaki Tanabe, Hideki Ohdan

**Affiliations:** a Department of Gastroenterological and Transplant Surgery, Graduate School of Biomedical and Health Sciences, Hiroshima University, 1-2-3, Kasumi, Minami-ku, Hiroshima 734-8551, Japan; b Department of Perioperative and Critical Care Management, Graduate School of Biomedical and Health Sciences, Hiroshima University, Hiroshima, Japan

**Keywords:** NIR, near-infrared, ICG, indocyanine green, US, ultrasound, GCLM, gastric cancer liver metastases, HCC, hepatocellular carcinoma, CK, cytokeratin, Indocyanine green, Near-infrared imaging, Hepatic metastasis, Gastric cancer, Case report

## Abstract

**Introduction and importance:**

Near-infrared (NIR) imaging with indocyanine green (ICG) is a sensitive intraoperative tool for detecting liver tumors. NIR imaging has been used to distinguish metastatic liver cancer from colon cancer; however, its utility for identifying metastatic lesions from gastric cancer remains unknown. We present a case of advanced gastric cancer with multiple liver metastases, which was diagnosed and treated using intraoperative NIR imaging with ICG.

**Case presentation:**

A 69-year-old man with advanced gastric cancer and simultaneous multiple liver metastases presented with gastric bleeding. He underwent gastrectomy and chemotherapy, which reduced the tumor burden. No new lesions were noted, and the patient was advised to undergo surgical resection of the residual liver metastases. Prior to surgery, 0.5 mg/kg of intravenous ICG was administered. NIR imaging was performed during hepatectomy, which revealed clear green fluorescence in several liver segments, indicating liver metastases. Two lesions were not identified during preoperative magnetic resonance imaging. All fluorescent areas were resected. The tumors identified by both preoperative and fluoresced intraoperatively demonstrated malignant features on histopathological examination. The two lesions that fluoresced intraoperatively but were not identified on preoperative images demonstrated normal liver parenchyma and no signs of malignancy. The patient remains tumor-free 1 year after surgery.

**Clinical discussion:**

This report demonstrates that hepatic metastases from gastric cancer can be diagnosed with intraoperative NIR imaging with ICG.

**Conclusions:**

NIR imaging with ICG can detect liver metastases but may provide false positive results. As the percentage of false positives is high, additional resections must be decided upon carefully.

## Highlights

• We used intraoperative NIR-ICG imaging to detect gastric cancer liver metastases.
• Two lesions fluoresced intraoperatively that were not identified preoperatively.
• Histopathology showed normal liver parenchyma and no malignancy in these lesions.
• NIR imaging with ICG detects liver metastases but may give false positive results, thus additional resections of liver tumor must be decided on carefully.

## 1 Introduction

Indocyanine green (ICG) is used to examine hepatic functional reserve [1], because the specific dynamics of ICG fluorescence can be utilized to evaluate blood flow and the liver parenchyma. ICG fluorescence can provide a variety of information on the biliary tract, including measurements of the hepatic area and assessment of blood flow. Near-infrared (NIR) imaging is often used in the clinical setting [2], [3]. After intravenous administration, ICG combines with serum proteins to form conjugated ICG, which is taken up by the liver and excreted into bile without being metabolized [4]. While NIR light is not usually visible, ICG fluorescence in tissues is clearly detected with NIR imaging, because ICG fluoresces following excitation by infrared light. NIR imaging with ICG can be used to identify liver tumors and metastases intraoperatively [5]. Here, we present a case of advanced gastric cancer with multiple liver metastases that was diagnosed and treated guided by intraoperative NIR imaging, which revealed false positive lesions, as confirmed by histopathological examination. This work has been reported in line with the SCARE criteria [6].

## 2 Presentation of case

A 69-year-old Japanese man with stage IV advanced gastric cancer with multiple liver metastases, classified as T3N1M1 according to the Union for International Cancer Control 8th edition, presented with gastric bleeding. Laboratory examination results revealed anemia, but all other findings were within the normal ranges. However, the patient's carcinoembryonic antigen levels were elevated at 7.1 mg/mL (normal range, <5 mg/mL).

Magnetic resonance imaging demonstrated four foci of metastasis in the posterior hepatic area (three in S6, and one in S7). In addition, one metastatic focus was documented in each of the following regions: the ventral region of S8, dorsal region of S5, and S3. The patient first underwent laparoscopic distal gastrectomy for gastric bleeding and then chemotherapy for liver metastases.

**Fig. 1:**
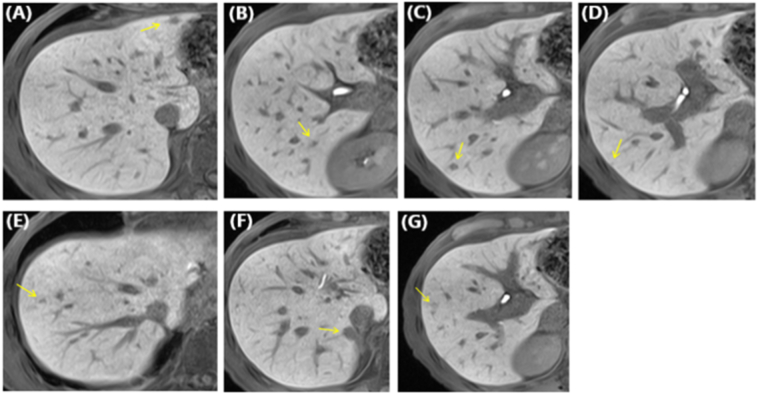
Ethoxybenzyl-enhanced magnetic resonance imaging findings following chemotherapy. The liver tumors (yellow arrows) in segments 3 (A), 5 (G), 6 (B, C, D), 7 (F), and 8 (E) are reduced. No new lesions are noted. (For interpretation of the references to colour in this figure legend, the reader is referred to the web version of this article.)

The patient tolerated three courses of chemotherapy but became anorexic and malnourished. Because the metastatic liver tumors decreased in size and no new metastatic lesions were noted (Fig. 1), chemotherapy was discontinued in preparation for radical conversion surgery. Comprehensively considering the number of metastatic tumors, location, liver functional reserve and general condition, radical surgical resection of the residual metastatic tumors in the liver was considered possible by gastroenterological surgeons at our institute.

**Fig. 2:**
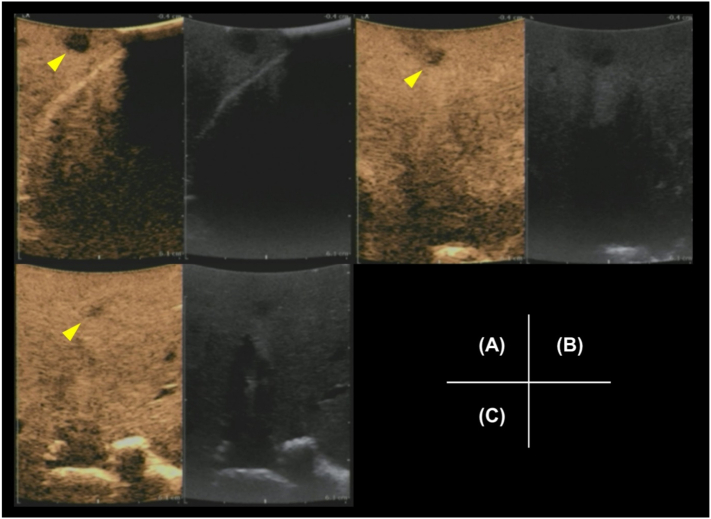
Intraoperative sonazoid-enhanced ultrasonography of the hepatic lesions. Lesions (yellow arrowheads) are noted in segments 3–1 (A), 5 (B), and 8 (C). No lesions are observed in segments 3–2 and 4. (For interpretation of the references to colour in this figure legend, the reader is referred to the web version of this article.)

**Fig. 3:**
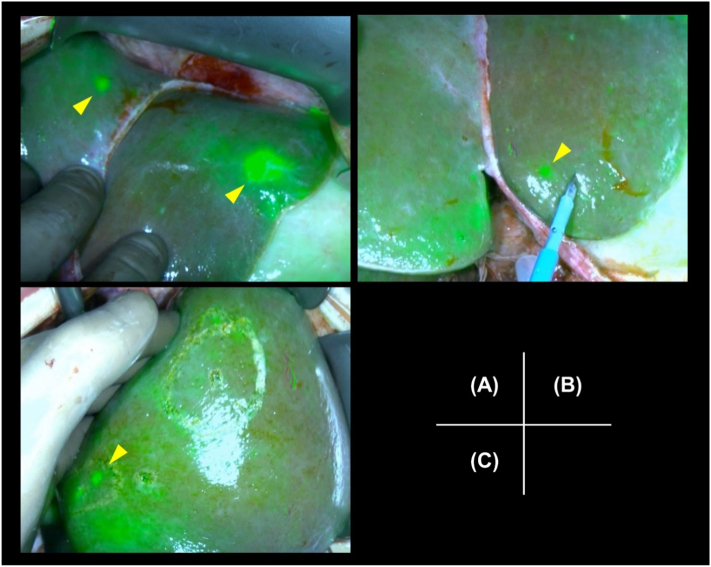
Intraoperative near-infrared imaging with indocyanine green of the hepatic lesions. (A) Lesions are identified in S3–1 (right yellow arrow) and S4 (left yellow arrow). (B) Partial excision of the fluorescent lesion in S3–2 is performed. (C) Two fluorescent lesions are noted in S5. (For interpretation of the references to colour in this figure legend, the reader is referred to the web version of this article.)

Intraoperatively, when we attempted to visualize the hepatic tumors that were to be partially resected (except the tumors in the posterior segment) with sonazoid-enhanced ultrasound (US) imaging, US detected the tumors in S3, S5, and S8 (Fig. 2) and no new lesions. To identify tumors that were undiagnosed on preoperative images, we elected to use NIR imaging (PINPOINT®, Novadaq, Mississauga, Canada) with 1 mL (0.5 mg/kg) of intravenous ICG. When we induced ischemia in the posterior segment of the liver with Glisson's capsule clamping, this method visualized and marked excision lines for systematic excision of the posterior area containing four tumors. Furthermore, the NIR imaging demonstrated two lesions in S3 (S3-1, S3-2 [preoperatively unknown]) and S5, and one lesion in S4 (preoperatively unknown) (Fig. 3). We partially resected the tumors in S3 (S3-1 and S3-2), S4, and S8 and performed extended posterior resection including the lesions in S5, S6, and S7. The extent of the resection was guided by ICG fluorescence, and all fluorescent areas were resected.

**Fig. 4:**
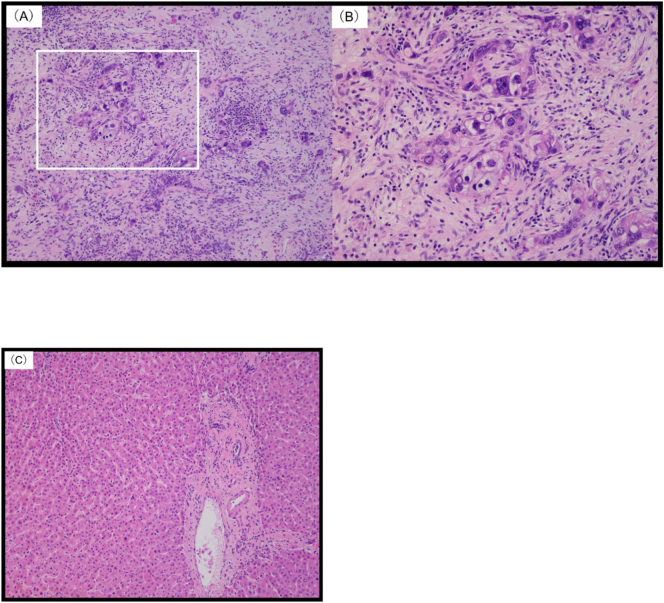
Histopathology findings of resected hepatic specimens. The specimens are stained with hematoxylin and eosin. (A) and (C) correspond to the lesions from S3–1 and S3–2, respectively. (B) shows the enlarged area marked by the white box in (A). (A) and (B) demonstrate atypical epithelial cells in glandular or vesicular patterns. (C) demonstrates normal hepatic lobular structures. The portal veins, bile ducts, and hepatic arteries are normal, but there is mild bile stasis. Regenerating nodules or fibrotic tissue are not found.

Histopathological analysis revealed metastatic tumors with atypical epithelial cells in glandular or vesicular patterns in the lesions in S3-1, S5, S6, S7, and S8. The hepatic specimens demonstrated infiltrating tumor tissue with fibrous stromal growth that was similar in histology to gastric cancer. Seven foci of metastases were noted, and all foci demonstrated the por2 histologic type. While foci in S3-2 and S4 (both preoperatively unknown) fluoresced with PINPOINT® (Novadaq) imaging, no evidence of neoplasia was documented in the specimens retrieved from these locations. No regenerating nodules or even fibrotic tissue were observed (Fig. 4). The patient remains tumor-free 1 year after surgery despite the absence of adjuvant therapy.

## 3 Discussion

Compared to colorectal liver metastases, the treatment criteria for gastric cancer liver metastases (GCLM), especially with regard to surgical resection, is less clear, and treatment is often individualized for most cases of GCLM. Over the last decade, the management of GCLM has shifted from chemotherapy alone to multidisciplinary treatment comprising chemotherapy and curative hepatectomy, because an increasing number of patients are responding to chemotherapy. Liver metastasectomy has recently been shown to improve long-term overall survival in patients with GCLM [7]. A systematic review by Montagnani et al. [8] proposed that surgical resection of liver metastases from gastric cancer provides significant improvements in 5-year and 10-year overall survival in some patients who demonstrate a response to systemic chemotherapy. A systematic review of the literature and meta-analysis examined the role of hepatectomy in GCLM and its associated prognostic factors, and demonstrated that well-moderately differentiated tumors, pT1–2 and pN0–1 tumors, solitary, unilobar, or metachronous metastasis, and R0 resection were associated with better overall survival and disease-free survival [9]. Thus, in cases where systemic chemotherapy for advanced gastric cancer with metastasis has been successful to some extent, the employment of a radical treatment strategy, known as conversion surgery, wherein localized or limited numbers of distant metastatic lesions are resected, is increasing.

In order to completely resect the hepatic metastases, it is important to clearly identify the tumors by using not only preoperative diagnostic imaging but also intraoperative imaging such as US imaging. However, it is often difficult to identify metastatic liver tumors that have shrunk after chemotherapy at the time of hepatic resection, even when utilizing intraoperative US. The usefulness of intraoperative identification of those liver tumors by ICG has already been reported for hepatocellular carcinoma (HCC), colorectal cancer liver metastases, hepatoblastoma liver tumors and many other solid cancers; however, there are no reports of intraoperative ICG tumor identification in GCLM. The most common uses of ICG for tumors are during cholangiography, segmentation mapping, and identification of liver lesions during hepatobiliary surgery [10]. ICG is a non-toxic organic anion that is taken up and released exclusively by mature hepatocytes into bile and then excreted, without undergoing biotransformation or entering the enterohepatic circulation, and is used clinically to test hepatic functions [11], [12]. In this case, we were able to detect all preoperatively identified and liver metastatic lesions by utilizing the characteristics of ICG uptake into tumors.

**Fig. 5:**
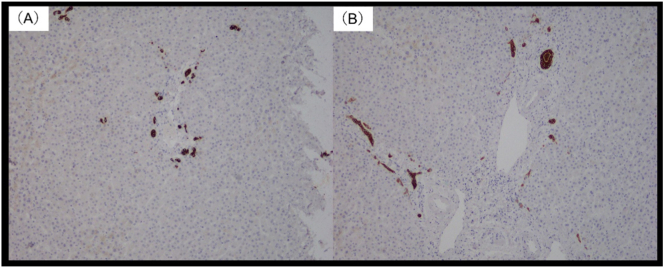
Immunohistochemistry staining of the resected non-metastatic tumor. The lesion from S3-2 is negative for cytokeratin (CK) 19 (A) and almost negative for CK 7 (B).

Notably, two lesions were identified by intraoperative NIR imaging with ICG but were later histopathologically confirmed to be false positive lesions. False positive results in NIR imaging with ICG in solid tumors, such as lung and ovarian cancer and HCC, have been reported; however, false positive results in metastatic liver tumors are rare [13], [14]. ICG uptake is mainly mediated by organ anion transporting polypeptide 1B3 and sodium/taurocholate *co*-transporting polypeptide, whereas ICG excretion is thought to be mediated mainly by multidrug resistance P-glycoprotein 3. Poorly differentiated HCC and colorectal liver metastases also exhibit tumor compression, which limits hepatocyte maturation around the tumor. Immature hepatocytes lack ICG excretion transporter expression, which results in ICG accumulation around the tumor and ring enhancement on NIR imaging. Compressed hepatocytes demonstrate increased ductular transformation, periportal fibrosis, and Kupffer cells. Immunohistochemical analysis has identified an association between fluorescence and cytokeratin (CK) 7 staining. CK7 is expressed by immature hepatocytes during ductular transformation [15]. We performed immunostaining of the immature hepatocytes in the sites that demonstrated ICG fluorescence but found no evidence of malignancy on histopathology. Immunostaining was negative for CK7 and CK19 (Fig. 5), which indicated that these sites had normal, non-weak hepatic parenchyma. Histopathological analysis of the specimens from the false positive areas demonstrated normal liver parenchyma in some samples but chronic inflammation and fibrosis, bile plug, liver cysts, and HCC in others [12]. However, NIR-ICG shows a significant number of false positives in liver cancer [16]. Therefore, resection of false positive lesions should be carefully considered.

## 4 Conclusion

We presented a case of hepatic metastases from gastric cancer diagnosed with intraoperative US and NIR imaging with ICG. Our findings support that the ICG fluorescence method is very useful for detecting liver metastases. However, the percentage of false positives is high, and thus additional resections of liver tumor which were not diagnosed preoperatively must be decided upon carefully.

## Ethics approval and consent to participate

Not applicable.

## Consent for publication

Written informed consent was obtained from the patient for publication of this case report and accompanying images. A copy of the written consent is available for review by the Editor-in-Chief of this journal on request.

## Availability of data and materials

The datasets supporting the conclusions of this article are included within the article.

## Research registration

Not applicable.

## Guarantor

Hiroyuki Tahara

## Provenance and peer review

Not commissioned, externally peer-reviewed.

## Author contributions

TT acquired the patient's data. TT and HT drafted the manuscript. All of the authors contributed to and edited the manuscript. TT, HT, YS, YY, NT, MH, SK, and MO managed the patient postoperatively. All authors read and approved the final manuscript.

## Funding

This research did not receive any specific grant from funding agencies in the public, commercial, or not-for-profit sectors.

## Declaration of competing interest

None.
